# Ciclopirox inhibits cancer cell proliferation by suppression of Cdc25A

**DOI:** 10.18632/genesandcancer.135

**Published:** 2017-03

**Authors:** Tao Shen, Chaowei Shang, Hongyu Zhou, Yan Luo, Mansoureh Barzegar, Yoshinobu Odaka, Yang Wu, Shile Huang

**Affiliations:** ^1^ Department of Biochemistry and Molecular Biology, Louisiana State University Health Sciences Center, Shreveport, LA, USA; ^2^ Feist-Weiller Cancer Center, Louisiana State University Health Sciences Center, Shreveport, LA, USA; ^3^ State Key Laboratory of Biotherapy / Collaborative Innovation Center of Biotherapy, West China Hospital, Sichuan University, Chengdu, Sichuan, P.R. China

**Keywords:** Ciclopirox, Cdc25A, cyclin dependent kinase, cell cycle, cell proliferation

## Abstract

Ciclopirox olamine (CPX), an off-patent fungicide, has recently been identified as a novel anticancer agent. However, the molecular mechanism underlying its anticancer action remains to be elucidated. Here we show that CPX inhibits cell proliferation in part by downregulating the protein level of Cdc25A in tumor cells. Our studies revealed that CPX did not significantly reduce Cdc25A mRNA level or Cdc25A protein synthesis, but remarkably promoted Cdc25A protein degradation. This resulted in inhibition of G_1_-cyclin dependent kinases (CDKs), as evidenced by increased inhibitory phosphorylation of G_1_-CDKs. Since Cdc25A degradation is tightly related to its phosphorylation status, we further examined whether CPX alters Cdc25A phosphorylation. The results showed that CPX treatment increased the phosphorylation of Cdc25A (S76 and S82), but only Cdc25A-S82A mutant was resistant to CPX-induced degradation. Furthermore, ectopic expression of Cdc25A-S82A partially conferred resistance to CPX inhibition of cell proliferation. Therefore, our findings indicate that CPX inhibits cell proliferation at least in part by promoting Cdc25A degradation.

## INTRODUCTION

Ciclopirox olamine (CPX), a synthetic fungicide, has been used for the treatment of superficial fungal infections for over two decades [1]. Studies have shown that CPX not only possesses antimycotic and antibacterial activities [1], but also arrests cells in G_1_ phase of the cell cycle in mammalian cells [2, 3]. A 4-week oral administration of CPX at 30 mg/kg body weight or a 3-month administration of CPX at 10 mg/kg does not exhibit obvious toxicity in various experimental animals [4]. Serum concentrations (∼10 μM) of CPX are achievable after repeated administration of CPX to rats and dogs, and are not toxic, suggesting favorable safety and pharmacokinetic profiles of CPX [5]. Recently, preclinical studies have revealed that CPX has potent anticancer activity in various cancer cell lines [6-16]. Also, a phase I clinical trial study has shown that oral administration of CPX at a dose of 40 mg/m^2^ once daily for 5 days is well tolerated in patients, and induces disease stabilization and/or hematologic improvement in 2/3 patients with advanced hematologic malignancies [17]. It has been shown that CPX exerts its anticancer activity by inhibiting proliferation, and inducing cell death in tumor cells [6,7]. In addition, CPX can also inhibit angiogenesis and lymphangiogenesis [18, 19]. Therefore, accumulating evidence suggests that CPX is a promising anticancer agent and may be repositioned for cancer therapy.

Despite the above exciting findings, the molecular mechanisms underlying the anticancer action of CPX are only at the beginning of investigation. It has been shown that CPX inhibits angiogenesis by blocking proline hydroxylation and maturation of collagen in human umbilical vein endothelial cells [18]. CPX inhibits lymphangiogenesis in an *in vitro* model (lymphatic endothelial cell tube formation) by suppressing vascular endothelial growth factor receptor 3 mediated extracellular signal-regulated protein kinases 1/2 signaling pathway [19]. CPX induces cell death in leukemia and myeloma cells by inhibiting the iron-dependent enzyme ribonucleotide reductase [6] and Wnt/β-catenin pathway [9]. CPX induces apoptosis in rhabdomyosarcoma and breast cancer cells by downregulating the protein levels of Bcl-xL and survivin and increasing the cleavage of Bcl-2 [7]. CPX induces autophagy by inducing reactive oxygen species and activating c-Jun *N*-terminal kinase cascade [14]. Of interest, CPX has been consistently found to inhibit cell proliferation by arresting cells in G_1_ phase of cell proliferation in a concentration-dependent manner, the cell cycle [3, 7, 15], which is attributed to inhibition of cyclin dependent kinases (CDKs) [7]. Nevertheless, how CPX inhibits CDKs is not fully elucidated.

The activities of CDKs are precisely regulated by multiple events including phosphorylation, dephosphorylation and protein-protein interactions, among which, the removal of inhibitory phosphorylation on CDKs by cell division cycle 25 (Cdc25), a dual-specificity protein phosphatase, is crucial for the full activation of CDKs [20-23]. The Cdc25 family has three members: Cdc25A, Cdc25B, and Cdc25C. Although the catalytic domains of these phosphatases are well conserved, their regulatory domains, which decide their subcellular distribution and turnover, are greatly diverse [22, 23]. While Cdc25B and Cdc25C promote G_2_/M progression by primarily dephosphorylating CDK1 at T14/Y15, two inhibitory phosphorylation sites [24, 25], Cdc25A plays a more extensive role in assisting both G_1_/S and G_2_/M progression by dephosphorylating CDK4 at Y17 [26], CDK6 at Y24 [27], as well as CDK2 and CDK1 at T14/Y15 [28 29]. More importantly, overexpression of Cdc25A has been frequently observed in multiple types of tumors, which is correlated to the poor prognosis in cancer patients [22]. Hence, Cdc25A has emerged as a potential target for cancer therapy [22]. Recently, we have noticed that CPX inhibits CDKs, in part by downregulating the protein levels of cyclins (A, B1, D1 and E) and CDK2/4 [7]. Given that Cdc25A is a very important positive regulator of CDKs, we hypothesized that Cdc25A may play a critical role in CPX-induced inhibition of CDK or inhibition of cell proliferation.

Here, for the first time, we show that Cdc25A is involved in CPX-induced inhibition of CDK or inhibition of cell proliferation. Our results indicated that CPX induced the phosphorylation of Cdc25A, which promoted protein degradation of Cdc25A in rhabdomyosarcoma (Rh30) and breast cancer (MDA-MB-231) cells. Downregulation of Cdc25 protein level consequently accumulated the inhibitory phosphorylation level on G_1_-CDKs. The finding sheds new insights on the mechanism by which CPX inhibits CDKs related to cell proliferation.

## RESULTS

### CPX inhibits cell proliferation in tumor cells

**Figure 1:**
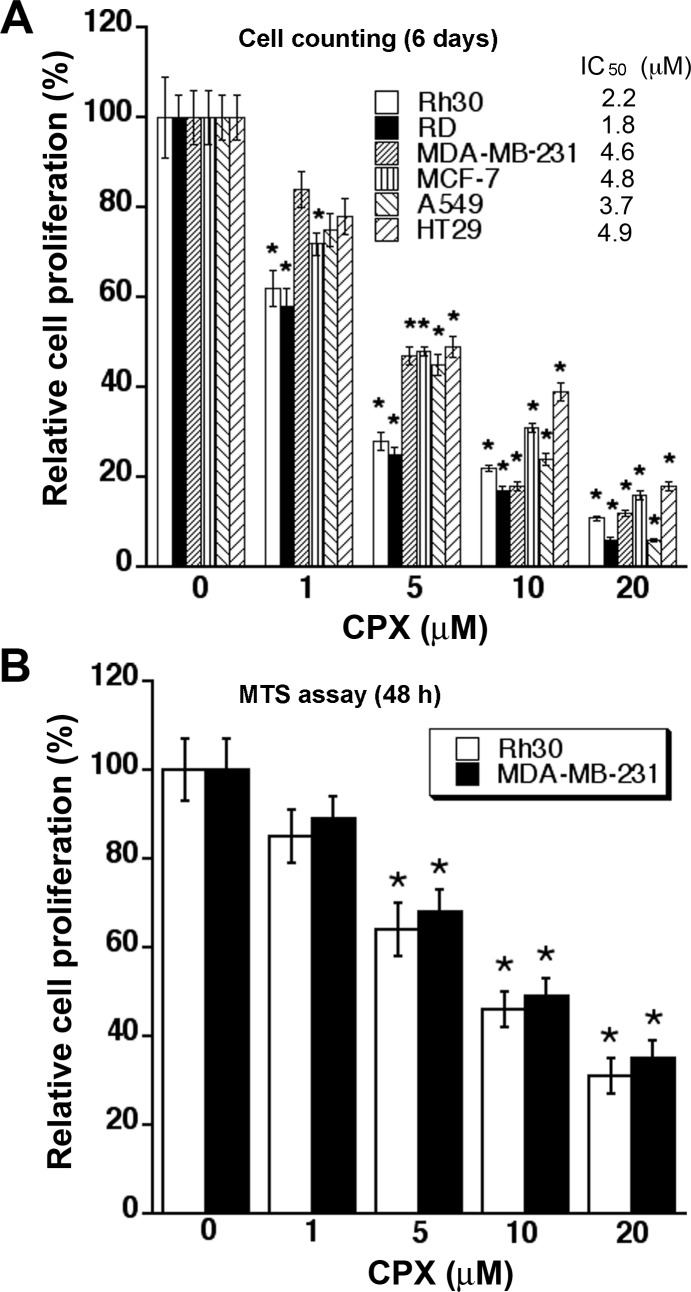
CPX inhibits cell proliferation in tumor cells (A) Indicated cell lines, grown under normal conditions, were treated with CPX (0-20 μM) for 6 days, followed by cell counting. (B) MDA-MB-231 and Rh30 cells were treated with CPX (0-20 μM) for 48 h, followed by one solution cell proliferation assay. All data represent the means ± SE (n=3). *P< 0.05.

To investigate the anti-proliferative effect of CPX on tumor cells, six human tumor cell lines, including Rh30 To investigate the anti-proliferative effect of CPX on tumor cells, six human tumor cell lines, including Rh30 and RD rhabdomyosarcoma cells, MDA-MB-231 and MCF7 breast cancer cells, A549 lung cancer cells, and HT29 colon cancer cells, were employed. When these cells, grown under normal growth conditions, were exposed to CPX (0-20 μM) for 6 days, CPX inhibited the cell proliferation in a concentration-dependent manner, with IC_50_ values ranging from 1.5 to 4.9 μM (Figure 1A). The results support the notion that CPX is a potent anticancer agent.

As MDA-MB-231 and Rh30 are both *p53* mutant [30, 31] and frequently used for cancer research, these two cell lines were selected for further experiments in this study. As detected by one solution assay, treatment with CPX for 48 h also inhibited proliferation of Rh30 and MDA-MB-231 cells in a concentration-dependent manner (Figure 1B). Of note, the 48-h growth inhibitory effect of CPX, particularly at higher concentrations (>10 μM), was not as potent as that in the above 6-day growth inhibition assay (Figure 1A). This is consistent with our previous findings that treatment with higher concentrations of CPX (10-20 μM) for 72 h or longer time not only inhibits cell proliferation, but also induces significant apoptosis in the tumor cells [7].

### CPX accumulates cells at G_1_ phase of the cell cycle

**Figure 2:**
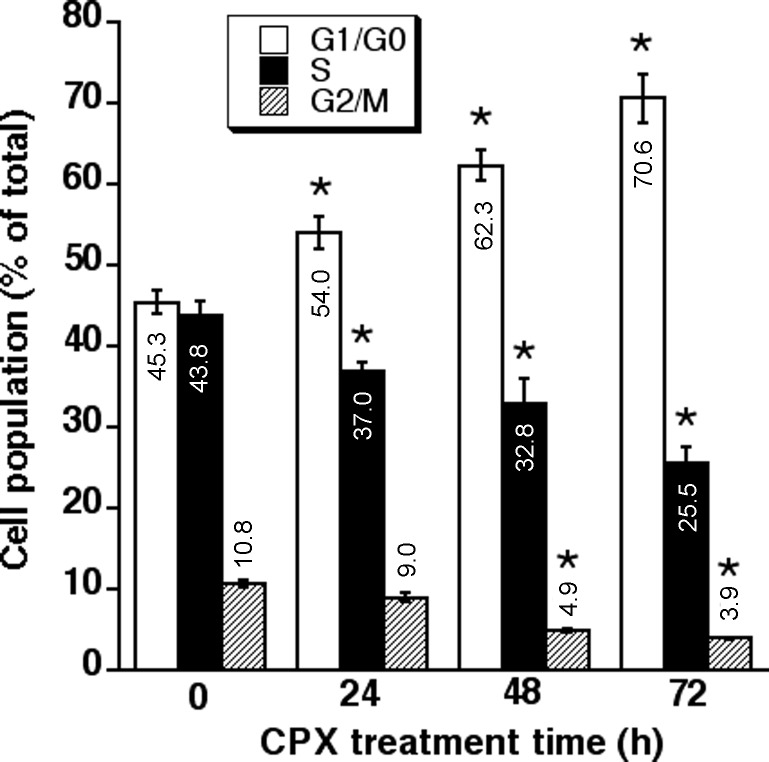
CPX induces accumulation of Rh30 cells at G_1_ phase of the cell cycle in a time-dependent manner Rh30 cells were exposed to CPX (0 and 5 μM) for 24, 48 and 72 h, respectively, followed by cell cycle analysis. All data represent the means ± SE (n=3). *P< 0.05.

Our previous dose-response experiments have shown that treatment with CPX (0-20 μM) for 24 h accumulates cells at G_1_/G_0_ phase in a concentration-dependent manner [7]. Since 5 μM of CPX was able to inhibit cell proliferation significantly in both MDA-MB-231 and Rh30 cells (Figure 1), this concentration was chosen for a time course analysis of the cell cycle, in order to determine whether CPX slows down cell cycle progression or arrests cells in G_1_ phase. As illustrated in Figure 2, CPX induced accumulation of Rh30 cells at G_1_/G_0_ phase in a time-dependent manner. Treatment with CPX (5 μM) for 24 h was able to significantly increase the G_1_ population. Correspondingly, the percentages of the cells in S and G_2_/M phases decreased. By extending the treatment for up to 72 h, which is longer than the doubling time (36 h) for Rh30 cell line [32], more cells were accumulated in G_1_/G_0_ phase, indicating that a G_1_ arrest was induced. Similarly, 24-h treatment with 5 μM of CPX also accumulated cells in G_1_ phase of the cell cycle in MDA-MB-231 cells (Supplementary Figure S1).

### CPX downregulates the expression of Cdc25A, resulting in increased inhibitory phosphorylation of G_1_-CDKs in tumor cells

**Figure 3:**
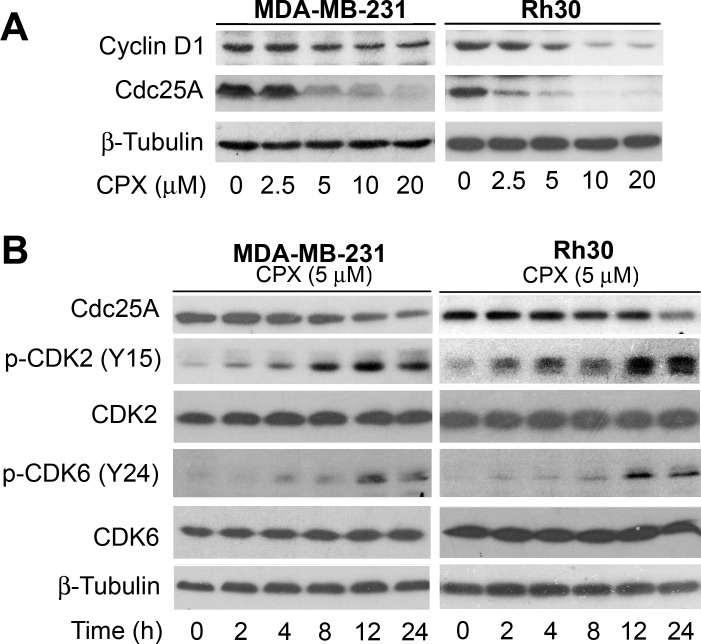
CPX downregulates the cellular protein level of Cdc25A, increasing the inhibitory phosphorylation level of CDK2 in tumor cells (A) MDA-MB-231 and Rh30 cells were treated with CPX (0-20 μM) for 24 h and 36 h, respectively, followed by Western blotting with indicated antibodies. (B) Indicated cells were treated with 5 μM of CPX for up to 24 h, followed by Western blotting with indicated antibodies.

Both cyclin D1-CDK4/6 and cyclin E-CDK2 complexes are important for G_1_→S cell cycle progression [20, 21]. Recently, we have observed that treatment with 5 μM of CPX for 24 h neither obviously downregulated the protein levels of cyclins A/B1 and CDK2, nor apparently upregulated the protein levels of the CDK inhibitors p21^Cip1^ and p27^Kip1^, but only modestly decreased the protein levels of cyclins D1/E and CDK4 [7]. Since Cdc25A is a positive regulator for G_1_-CDKs [22, 23], we speculated that 5 μM of CPX may induce the G_1_ arrest of the cells by decreasing Cdc25A level. To this end, MDA-MB-231 cells were treated with CPX (0-20 μM) for 24 h, followed by Western blot analysis. We found that treatment with CPX (0-20 μM) for 24 h reduced the levels of Cdc25A in a concentration-dependent manner (Left panel, Figure 3A). Of interest, treatment with CPX (≥5 μM) for 24 h was able to dramatically reduce the level of Cdc25A in the cells. In contrast, the inhibitory effect of CPX on cyclin D1 expression was modest (Left panel, Figure 3A). Similar results were observed in Rh30 cells (Right panel, Figure 3A). Moreover, in A549 and HT29 cells, 24-h treatment with CPX did not obviously downregulate cyclin D1 protein level, but markedly reduced Cdc25A protein level in a concentration-dependent manner (Supplementary Figure S2). The results support our hypothesis that CPX downregulation of Cdc25A may play a critical role in inducing G_1_ cell cycle arrest.

Since studies have demonstrated that CDK2 and CDK4/6 have overlapped functions [20, 21], it is necessary to inhibit both D1-CDK4/6 and E-CDK2 for completely arresting cells in G_1_ phase. Having observed that treatment with 5 μM of CPX significantly induced G_1_ arrest (Figure 2) and remarkably downregulated the protein level of Cdc25A in tumor cells (Figure 3A), we reasoned that CPX at this concentration might inhibit the activity of CDK2 by increasing the level of its inhibitory phosphorylation, leading to G_1_ arrest in the cells. For this, MDA-MB-231 cells were treated with CPX (5 μM) for 0-24 h, followed by Western blot analysis. As predicted, treatment with CPX (5 μM) reduced the protein level of Cdc25A in a time dependent manner. Concurrently, CPX increased the level of the inhibitory phosphorylation of CDK2 (Y15) in the cells (Left panel, Figure 3B), indicating inhibition of CDK2 activity. Similar data were observed in Rh30 cells (Right panel, Figure 3B). Since Cdc25A can dephosphorylate both CDK2 and CDK4/6 [22, 23], we also checked whether CPX induces the inhibitory phosphorylation of CDK6. As expected, treatment with CPX (5 μM) also induced the inhibitory phosphorylation of CDK6 (Y24) in both MDA-MB-231 and Rh30 cells in a time dependent manner (Figure 3B). Collectively, the results indicate that CPX downregulation of Cdc25A protein level contributes to the inhibition of G_1_-CDKs, resulting in the G_1_ cell cycle arrest.

### CPX downregulates Cdc25A expression by promoting Cdc25A protein degradation

**Figure 4:**
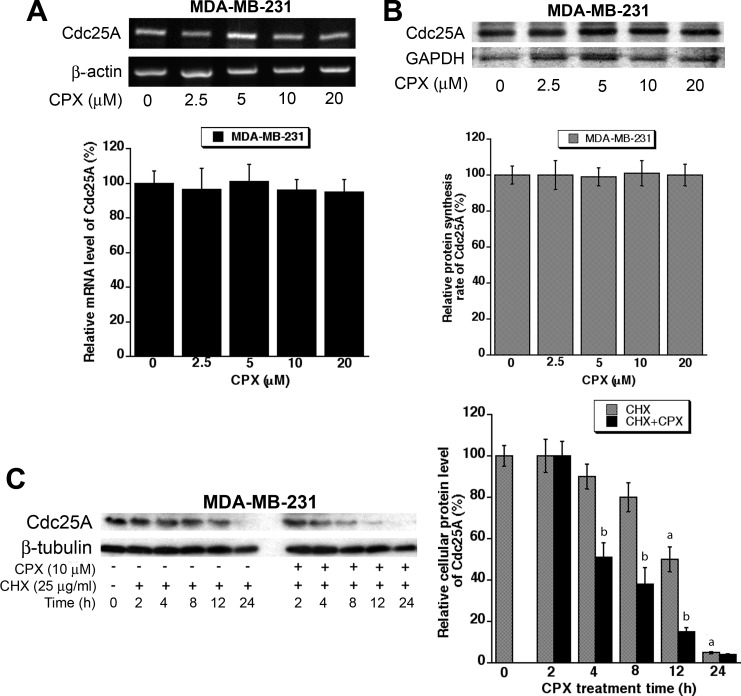
CPX does not reduce Cdc25A mRNA level or protein synthesis significantly, but promotes Cdc25A protein degradation markedly (A) MDA-MB-231 cells were treated with CPX (0-20 μM) for 24 h. Total mRNA was extracted, followed by RT-PCR. β-actin was used as internal control. (B) MDA-MB-231 cells were treated with CPX at 0-20 μM for 18 h, and then pulsed with ^35^S-Met/Cys for 6 h in the presence of CPX (0-20 μM). The proteins in the cell lysates were subjected to SDS-PAGE and transferred to a PVDF membrane, followed by autoradiography. GAPDH served as a control. (C) MDA-MB-231 cells were treated with 25 μg/ml cycloheximide (CHX), in the presence or absence of 10 μM CPX, for 0-24 h, followed by Western blotting with indicated antibodies. β-tubulin was used as a loading control. NIH Image J was used for semi-quantitative analysis of the intensities of the bands in (A-C). Results are means ± SE and are pooled from three independent experiments. ^a^*P* < 0.05, difference *versus* control group. ^b^*P* < 0.05, difference *versus* CHX group.

Regulation of Cdc25A protein level may occur at transcriptional, translational and/or posttranslational level [22, 23]. To elucidate how CPX downregulates the protein level of Cdc25A, semi-quantitative RT-PCR was first employed to determine whether CPX reduces Cdc25A mRNA expression. As shown in Figure 4A, treatment with CPX (2.5-20 μM) for 24 h slightly but not significantly reduced Cdc25A mRNA level in MDA-MB-231 cells. Similar results were observed in Rh30 cells (Supplementary Figure S3A).

Next, ^35^S-Met/Cys labeling was used to determine whether CPX downregulates Cdc25A protein expression by decreasing Cdc25A protein synthesis. For this, MDA-MB-231 were pretreated with CPX at 0-20 μM for 18 h, and then pulsed with ^35^S-Met/Cys 6 h in the presence of CPX (0-20 μM), followed by autoradiography. The results indicate that pretreatment with CPX at 2.5-20 μM for 18 h did not obviously inhibit incorporation of ^35^S-Met/Cys into Cdc25A, compared with the control (Figure 4B). Similar results were observed in Rh30 cells (Supplementary Figure S3B).

To determine whether CPX downregulates Cdc25A protein expression by increasing its protein degradation, MDA-MB-231 cells were exposed to 25 μg/ml of cycloheximide (CHX), an inhibitor of eukaryotic protein synthesis by preventing initiation and elongation on 80S ribosomes [33], in the presence or absence of CPX (10 μM) for up to 24 h, followed by Western blot analysis. It turned out that CPX treatment strikingly promoted the protein turnover rate of Cdc25A. As illustrated in Figure 4C, approximately 50% of Cdc25A protein was still detectable when the cells were treated with CHX alone for 12 h. However, about 50% of Cdc25A protein was observed when the cells were treated with CHX + CPX only for 4 h. Taken together, our results indicate that CPX altered neither mRNA level nor protein synthesis of Cdc25A, but promoted protein degradation of Cdc25A, thereby downregulating cellular protein expression of Cdc25A in the tumor cells. Similar results were observed in Rh30 cells (Supplementary Figure S3C).

It is well known that the ubiquitin/proteasome pathway plays an important role in Cdc25A degradation [41-47]. To substantiate whether CPX-induced degradation of Cdc25A is through proteasome-dependent mechanism, MG132 (a proteasome inhibitor) was used. As expected, co-treatment with MG132 (10 μM) did strongly prevent CPX from reducing the protein level of Cdc25A (see Supplementary Figure S4). The result support that CPX induced the protein degradation of Cdc25A indeed by ubiquitin-proteasome pathway.

### CPX-induced phosphorylation of Cdc25A links to its degradation

**Figure 5:**
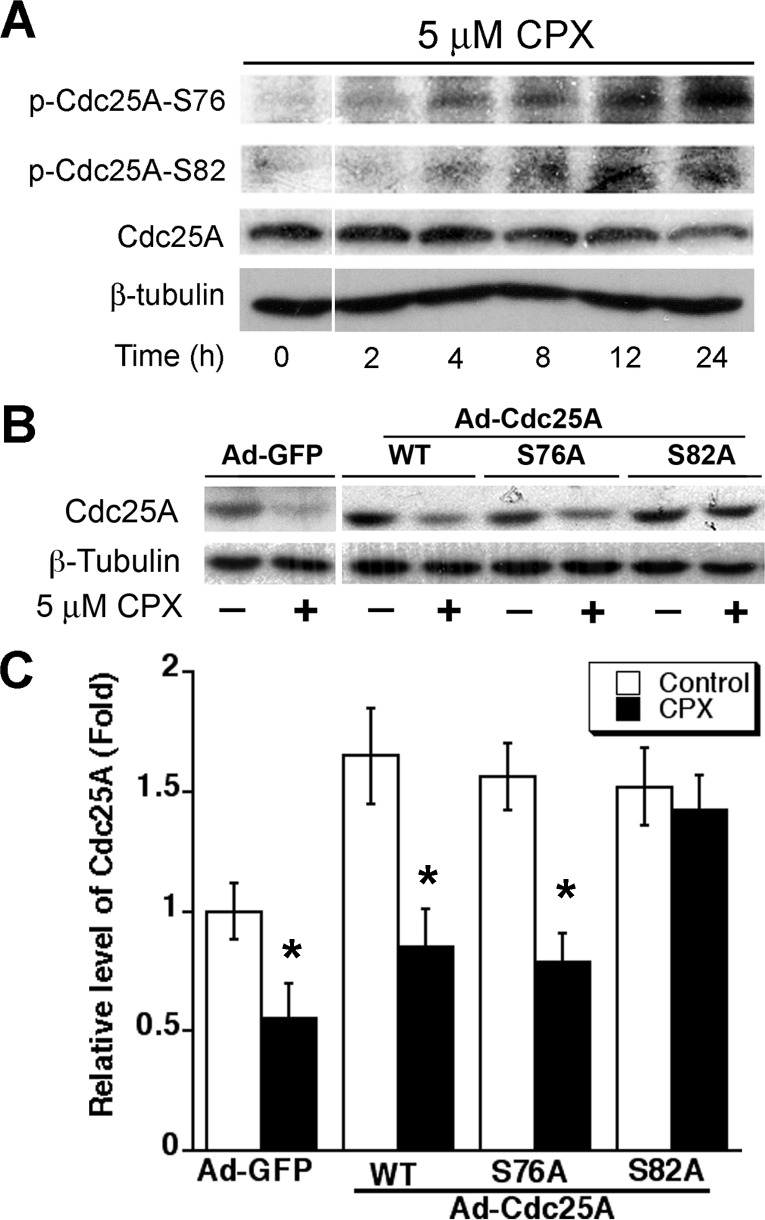
CPX-induced Cdc25A phosphorylation contributes to its degradation (A) Rh30 cells were treated with CPX (5 μM) for 0-24 h, followed by Western blotting with indicated antibodies. (B) Rh30 cells, infected with indicated recombinant adenoviruses, were treated with or without CPX (5 μM) for 24 h, followed by Western blotting with indicated antibodies. β-tubulin was used as a loading control. (C) Semi-quantitative analysis of the intensities of the bands in (B) using NIH Image J. All data represent the means ± SE (n=3). *P< 0.05.

As Cdc25A degradation is tightly associated with its phosphorylation [22, 23], next, we examined the phosphorylation status of Cdc25A. As shown in Figure 5A, treatment with CPX (5 μM) for 0-24 h accumulated the phosphorylation on Cdc25A (S76 and S82) in Rh30 cells in a time-dependent manner. The cellular protein level of Cdc25A was reversely correlated to the phosphorylation status of Cdc25A. Similar data was observed in MDA-MB-231 cells (Supplementary Figure S5A). The results suggest that CPX might induce the phosphorylation of Cdc25A, leading to the degradation of Cdc25A.

To corroborate whether CPX-induced phosphorylation of Cdc25A really contributes to its degradation, recombinant adenoviruses expressing GFP, wt or mutant Cdc25A (S76A or S82A) were generated and used to infect Rh30 cells. Of interest, infection with Ad-Cdc25A-wt, Ad-Cdc25A-S76A or Ad-Cdc25A-S82A upregulated the Cdc25A protein levels by 1.5∼1.8 fold, compared to infection with Ad-GFP. Ectopic expression of Cdc25-S82A, but not GFP, Cdc25A-wt, or Cdc25A-S76A, conferred significant resistance to CPX-induced degradation of Cdc25A (Figures 5B and 5C). Similar results were observed in MDA-MB-231 cells (Supplementary Figure S5B). The results indicate that CPX-induced degradation of Cdc25A is at least related to its induction of Cdc25A phosphorylation on S82A.

### Ectopic expression of Cdc25A mutant (S82A) renders resistance to CPX inhibition of cell proliferation

**Figure 6:**
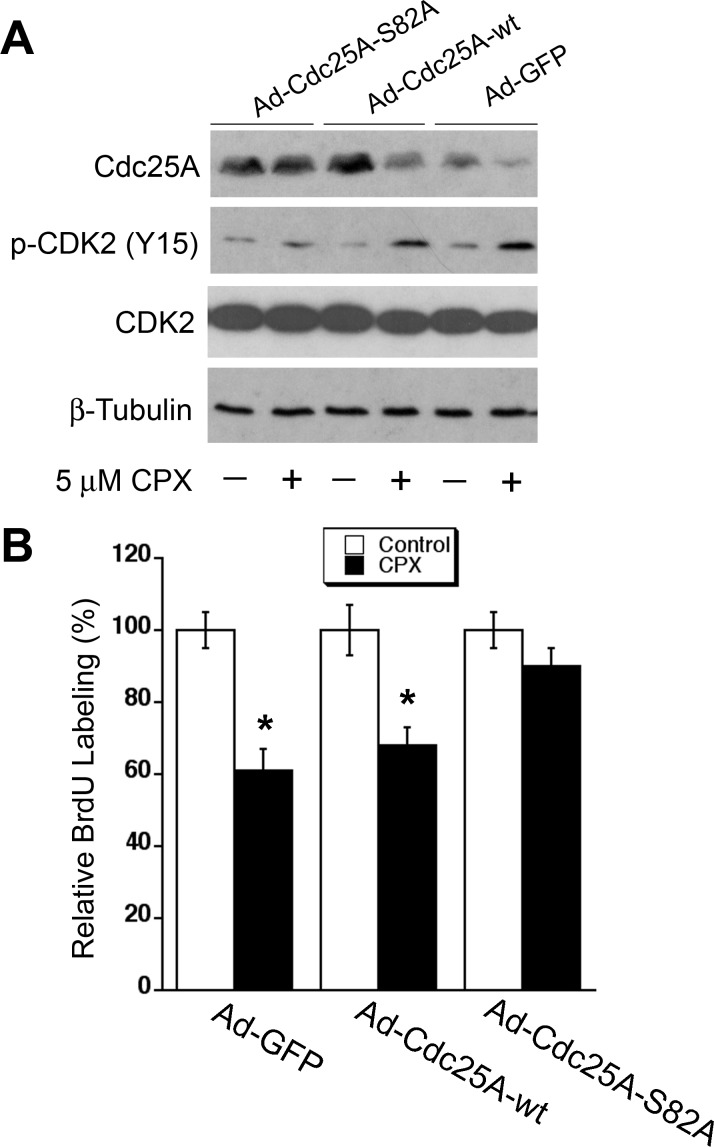
Ectopic expression of Cdc25A mutant (S82A) renders resistance to CPX inhibition of cell proliferation (A, B) Rh30 cells, infected with indicated recombinant adenoviruses, were treated with 5 μM of CPX for 36 h, followed by Western blotting with indicated antibodies (A), and BrdU labeling (B). All data represent the means ± SE (n=3). *P< 0.05.

Since ectopic expression of Cdc25A-S82A mutant conferred high resistance to CPX-induced degradation of Cdc25A (Figure 5B), we asked whether expression of this mutant affects CPX-induced CDK2 phosphorylation. Consistent with the above results (Figure 5), infection with Ad-Cdc25A-S82A or Ad-Cdc25A-wt upregulated the Cdc25A protein levels by 1.5∼2 fold, compared to infection with Ad-GFP. Treatment with CPX (5 μM) reduced Cdc25A levels by approximately 50% in the cells infected with Ad-GFP or Ad-Cdc25A-wt, but did not apparently alter Cdc25A levels in the cells infected with Ad-Cdc25A-S82A. As expected, CPX increased the levels of p-CDK2 (Y15) in Ad-GFP or Ad-Cdc25A-wt infected cells by about 2-fold, but only very slightly in Ad-Cdc25A-S82A infected cells (Figure 6A). In line with this, ectopic expression of Cdc25A-S82A, but not GFP or Cdc25A-wt, also rendered cells resistant to CPX-induced inhibition of cell proliferation, as assessed by BrdU labeling (Figure 6B). Similar results were observed in MDA-MB-231 cells (see Supplementary Figure S6). Collectively, our results demonstrate that CPX inhibits cell proliferation by inducing the phosphorylation of Cdc25A on S82, leading to the degradation of Cdc25A and inhibition of CDK2.

## DISCUSSION

CPX is an off-patent fungicide, which has been used to treat fungal infection of the skin and nails for more than 20 years [1]. Recent studies have demonstrated that CPX also possesses anti-proliferative activity in tumor cells [6, 7]. It has been described that CPX inhibits DNA synthesis by suppressing ribonucleotide reductase [6]. Also, CPX, at high concentrations (>10 μM), downregulates the cellular protein levels of cyclins (A, B1, D1 and E) and cyclin-dependent kinases (CDK2 and CDK4), and upregulates the protein level of the CDK inhibitor (p21^Cip1^) [7]. Here, for the first time, we show that CPX, at low concentrations (≤5 μM), also reduced the cellular protein level of Cdc25A, which resulted in increased inhibitory phosphorylation of G_1_-CDKs, leading to the accumulation of cells in G_1_ phase of the cell cycle in breast carcinoma (MDA-MB-231) and rhabdomyosarcoma (Rh30) cells. Taken together, the findings from this group and others support the notion that CPX inhibits tumor cell proliferation by multiple mechanisms, and Cdc25A is one of the critical targets of CPX.

As Cdc25A is important for cell cycle progression, its activity has to be timely and precisely regulated during the whole cell cycle. This can be achieved by multiple mechanisms including the regulation of Cdc25A expression at transcriptional [34, 35], translational [36, 37], and/or post-translational levels [38], as well as the regulation of the catalytic efficiency of Cdc25A by modulating its phosphatase activity [36] and enzyme-substrate interaction [39, 40]. In this study, we found that CPX downregulated the cellular protein level of Cdc25A at the post-translational level. This is strongly supported by our results that CPX did not significantly reduce the mRNA level of Cdc25A or the protein synthesis rate of Cdc25A, but remarkably promoted the protein degradation rate of Cdc25A in tumor cells (Figure 4 and Supplementary Figure S3). CPX-induced degradation of Cdc25A was proteasome-dependent, as treatment with MG132, a proteasome inhibitor, almost dramatically blocked the reduction of Cdc25A protein level triggered by CPX (Supplementary Figure S4). In addition, we observed that CPX-induced Cdc25A degradation was closely related to induction of Cdc25A phosphorylation on S82, as ectopic expression of the mutant Cdc25A-S82A, but not Cdc25A-wt or Cdc25A-S76A, conferred high resistance to CPX-induced degradation of Cdc25A in Rh30 (Figure 5) and MDA-MB-231 cells (Supplementary Figure S5). Furthermore, we found that ectopic expression of Cdc25A-S82A rendered cells resistant to CPX-induced CDK2 phosphorylation (Y15) and cell proliferation inhibition in Rh30 (Figure 6) and MDA-MB-231 cells (Supplementary Figure S6).

A derived question from the current work is the identity of the kinase that phosphorylates Cdc25A, leading to its degradation. It has been described that Chk1 phosphorylates multiple residues on Cdc25A, among which, phosphorylation on S76 is necessary for further phosphorylation on S79 and S82, residues locating in DSG motif (RMGS76SES79TDS82GFCLDS88PGPLD), by casein kinase 1 [41-46]. After phosphorylation at S82, an E3 ligases complex, SCF (Skp1/Cul1/F-box protein) binds to Cdc25A through interaction between DSG motif and β-TrCP (F-box protein) to facilitate Cdc25A ubiquitination and subsequent degradation [41, 47]. In addition, both glycogen synthase kinase 3β (GSK-3β) and serine-threonine kinase 38 (STK38) can also phosphorylate S76 residue of Cdc25A [48, 49]. The GSK-3β-induced phosphorylation on S76, which requires a prime phosphorylation on T80 by Polo-like kinase 3, is associated with SCF-mediated Cdc25A degradation and G1/S cell cycle arrest [48]. Recent studies have further shown that serine/threonine protein kinase SMG1 mediates the degradation of Cdc25A by directly phosphorylating Cdc25A in a Chk1-independent manner [50], though a phosphorylation site has not been identified. In our study, we noticed co-treatment with GSK-3β inhibitor (10 mM LiCl) for 24 h did not prevent CPX from inducing Cdc25A degradation in MDA-MB-231 cells, suggesting that GSK-3β might not be the kinase that phosphorylates Cdc25A, leading to Cdc25A degradation, in response to CPX treatment. In addition, we found that although CPX induced the phosphorylation of both S76 and S82 (Figure 5A), CPX reduced the Cdc25A protein level in the cells expressing the S76A mutant, but not the S82A mutant (Figure 5B), further implying that the kinase that phosphorylates S82 may not require a prior phosphorylation on S76. Clearly, more studies are required to identify the CPX-activated kinase that phosphorylates Cdc25A.

In summary, here we have shown that treatment with CPX induced the phosphorylation of Cdc25A, which promoted the protein degradation of Cdc25A in rhabdomyosarcoma (Rh30) and breast cancer (MDA-MB-231) cells. Downregulation of Cdc25A protein expression resulted in accumulation of the inhibitory phosphorylation of G_1_-CDKs, thus interrupting the cell cycle progression. The findings provide a new insight into the molecular mechanism by which CPX inhibits CDKs related to cell proliferation.

## MATERIALS AND METHODS

### Materials

Ciclopirox olamine (CPX) (Sigma, St. Louis, MO, USA) was dissolved in 100% ethanol to prepare a stock solution (100 mM), then aliquoted and stored at-20°C. RPMI 1640, Dulbecco's Modifid Eagle Medium (DMEM), and DMEM/F-12 were purchased from Mediatech (Herndon, VA, USA). Fetal bovine serum (FBS) was from Hyclone (Logan, UT) and 0.05% Trypsin-EDTA was from Invitrogen (Grand Island, NY, USA). 5-bromo-2-deoxyuridine (BrdU) and 4′,6-diamidino-2-phenylindole (DAPI) were from Life Technologies (Grand Island, NY, USA). Enhanced chemiluminescence solution was obtained from PerkinElmer Life Science (Boston, MA, USA). The following primary antibodies were used, including those against BrdU (Cat.# sc-32323), cyclin D1 (Cat.# sc-20044), CDK6 (Cat.# sc-56282), p-CDK6 (Y24) (Cat.# sc-293097), CDK2 (Cat.# sc-6248), Cdc25A (Cat.# sc-97), GAPDH (Cat.# sc-32233) (Santa Cruz Biotechnology, Santa Cruz, CA, USA), p-CDK2 (Y15) (Cat.# ab76146), p-Cdc25A (S76) (Cat.# ab75743) (Abcam, Cambridge, MA, USA), p-Cdc25A (S82) (a gift from Dr. Helen Piwnica-Worms, Washington University School of Medicine, St. Louis, MO) [43], and β-tubulin (Cat.# T4026) (Sigma, St. Louis, MO, USA). Goat anti-mouse IgG-horseradish peroxidase (Cat.# 31430) and goat anti-rabbit IgG-horseradish peroxidase (Cat.# 31460) were purchased from Pierce (Rockford, IL, USA). All other reagents were obtained from Sigma-Aldrich (St. Louis, MO, USA) unless otherwise specified.

### Cell lines and cultures

Human rhabdomyosarcoma (Rh30 and RD) cells were generously provided by Dr. Peter J. Houghton (St. Jude Children's Research Hospital, Memphis, TN, USA), and were grown in antibiotic-free RPMI 1640 medium supplemented with 10% FBS. Human breast carcinoma (MDA-MB-231 and MCF7), colorectal adenocarcinoma (HT29), and lung carcinoma (A549) cells were purchased from American Type Culture Collection (Manassas, VA, USA). MDA-MB-231 and MCF7 cells were grown in antibiotic-free DMEM/F12 supplemented with 10% FBS, while HT29 and A549 were cultured in antibiotic-free DMEM supplemented with 10% FBS. All cell lines were cultured in a humidified incubator (37°C and 5% CO_2_). In all treatments, CPX was dissolved in 100% ethanol to prepare a stock solution (100 mM). The subconfluent cells (60-70% confluent) were treated with various concentrations of CPX in the complete cell culture medium. Cells treated with vehicle (ethanol, final concentration in media = 0.1%) served as a control.

### Recombinant adenoviral constructs and infection

To generate recombinant adenoviruses expressing Cdc25A-wild type (wt) or mutants, DNA encoding wt or mutant Cdc25A (S76A or S82A) was, respectively, excised from pCDNA4/TO-Cdc25A-wt, S76A, or S82A [51] (gifts from Dr. Xianghong Zou, Ohio State University, Columbus, OH, USA) using BamH1 and EcoR1. The recombinant adenoviruses and the control virus expressing green fluorescence protein (GFP) were generated and amplified using ViraPower^™^ Adenoviral Gateway^™^ Expression Kit (Invitrogen, Carlsbad, CA, USA) following the manufacture's instruction. All adenoviruses were amplified, titrated and used as described previously [52, 53].

### Cell proliferation assay

Cell proliferation was evaluated using either CellTiter 96^®^ AQ_ueous_ One Solution Cell Proliferation Assay kit (Promega, Madison, WI, USA) or direct cell counting using a Beckman Coulter Counter (Beckman Coulter, Fullerton, CA, USA), as described previously [7].

### BrdU labeling

Rh30 cells, infected with Ad-GFP, Ad-Cdc25A-wt, -S76A, or -S82A, were plated on the coverslips in 6-well plates. The next day, the cells were treated with or without 5 μM of CPX for 24 h, followed by labeling with 3 μg/ml of BrdU for 3 h at 37°C. The cells were then fixed with cold 70% ethanol for 10 min at room temperature, rinsed 3 times with phosphate buffered saline (PBS), and treated with 2N HCl for 30 min at room temperature to denature DNA. Next, the cells were rinsed 3 times with PBS, and incubated with 5% normal horse serum (diluted in PBS containing 0.2% Triton X-100) for 1 h at room temperature to block nonspecific epitopes. Afterwards, the cells were incubated with mouse monoclonal anti-BrdU antibody overnight at 4°C. Following rinsing 3 times with PBS, the cells were further incubated with goat anti-mouse IgG conjugated with Texas Red (Santa Cruz Biotechnology, Santa Cruz, CA, USA) for 1 h at room temperature in dark. The cells were rinsed 3 times with PBS, and then further stained with 1 μg/ml DAPI (dissolved in Milli H_2_O) for 3 min at room temperature. Following a brief washing with PBS, slides were mounted in glycerol/PBS (1:1, v/v) containing 2.5% 1,4-diazabiclo-(2,2,2)octane. Photographs for BrdU (red) and DAPI (blue) staining were taken under a Nikon Eclipse TE300 fluorescence microscope equipped with a digital camera. The proliferation index was determined by measuring the percentage of BrdU positive cells. At least 500 cells were counted.

### Cell cycle analysis

Cell cycle analysis was performed, as described previously [7]. Briefly, Rh30 or MDA-MB-231 cells were treated with CPX at 5 μM for 0-72 h, followed by staining with the Cellular DNA Flow Cytometric Analysis Kit (Roche Diagnostics, Indianapolis, IN, USA). Percentages of cells within each of the cell cycle compartments (G_0_/G_1_, S, or G_2_/M) were determined using a FACSCalibur flow cytometer (Becton Dickinson, Mountain View, CA, USA) and ModFit LT analyzing software (Verity Software House, Topsham, ME, USA). Cells treated with vehicle alone (100% ethanol) were used as a control.

### Reverse transcription-polymerase chain reaction (RT-PCR) analysis for Cdc25A mRNA expression

Total mRNA was extracted from Rh30 or MDA-MB-231 cells, after treatment with CPX (0-20 μM) for 24 h, using TRIzol reagent (Invitrogen) following the manufacturer's instruction. cDNA was made using MML-VII reverse transcriptase (Invitrogen) and oligo (dT)_12–18_ primer (Invitrogen). PCR was performed using 10 ng cDNA, Taq DNA polymerase (New England Biolabs, Ipswich, MA, USA) and specific primer pairs for Cdc25A and β-actin (as internal control), respectively. The forward primer for Cdc25A is: 5′-atggaactgggcccggag-3′; and the backward primer is: 5′-agtggctgtcacaggtgact-3′; the forward primer for β-actin is: 5′-TACGGGGTCACCCACACTGTGCCCAT-3; and the backward primer is: 5′-CTAGAAGCATTTGCGGTGGACGATGGAGGG-3′. The amplification was done for 30 cycles (94°C 30s, 62°C 30 s and 72°C 40 s). PCR products were size fractionated on 2% agarose gels and photographed on a UV transilluminator equipped with a digital camera. NIH Image J (https://imagej.nih.gov/ij/) was used for semi-quantitative analysis of the intensities of the bands.

### ^35^S-Met/Cys labeling for Cdc25A protein synthesis

^35^S-Met/Cys labeling was performed, as described previously [54]. Briefly, Rh30 or MDA-MB-231 cells, grown in 100-mm dishes to 70% confluence, were treated with CPX (0-20 μM) for 30 h or 18 h, respectively. Subsequently, the cells were briefly washed with PBS twice, and cultured in 3 ml labeling medium (DMEM, without L-Met/L-Cys, Mediatech, Herndon, VA, USA) containing 10 μM MG-132 for 10 min to deplete Met/Cys in the cells, in the presence of CPX (0-20 μM). The cells were then pulsed with 0.3 mCi ^35^S-Met/Cys (MP Biomedicals, Solon, OH, USA) for 6 h in the presence of CPX (0-20 μM), and lysed in the RIPA buffer [50 mM Tris, pH 7.2; 150 mM NaCl; 1% sodium deoxycholate; 0.1% SDS; 1% Triton-X 100; 10 mM NaF; 1 mM Na_3_VO_4_; protease inhibitor cocktail (1:1000, Sigma, St. Louis, MO, USA)], followed by immunoprecipitation with antibodies to Cdc25A and GAPDH, respectively. The immunocomplexes were subjected to SDS-PAGE, transferred to a PVDF membrane, and finally autoradiographed at −80°C. NIH Image J was used for semi-quantitative analysis of the intensities of the bands.

### Cdc25A protein degradation assay

To determine the effect of CPX on Cdc25A protein degradation, Rh30 or MDA-MB-231 cells, grown in the growth medium to 70% confluence, were treated with cycloheximide (CHX) (25 μg/ml for MDA-MB-231 cells, and 50 μg/ml for Rh30 cells), in the presence or absence of CPX (10 μM), for 0-24 h, followed by Western blotting with antibodies to Cdc25A and β-tubulin (loading control), respectively. NIH Image J was used for semi-quantitative analysis of the intensities of the bands.

### Western blotting

Western blotting was performed as described previously [7]. NIH Image J was used for semi-quantitative analysis of the intensities of the bands.

### Statistical analysis

Results were expressed as mean values ± standard error (mean ± SE). The data were analyzed by one-way analysis of variance (ANOVA) followed by post-hoc Dunnett's *t*-test for multiple comparisons. A level of *P* < 0.05 was considered to be statistically significant.

## SUPPLEMENTARY MATERIAL FIGURES



## ABBREVIATIONS

BrdU: 5-bromo-2-deoxyuridine
Cdc25: cell division cycle 25
CDK: cyclin dependent kinase
CPX: ciclopirox olamine
DAPI: 4′,6-diamidino-2-phenylindole
DMEM: Dulbecco's Modifid Eagle Medium
FBS: Fetal bovine serum
GFP: green fluorescence protein
GSK-3β: glycogen synthase kinase 3β
PBS: phosphate buffered saline
RT-PCR: reverse transcription-polymerase chain reaction.
