# Supplementary material for: Ciclopirox inhibits cancer cell proliferation by suppression of Cdc25A
Source: Genes Cancer. 2017 Mar;8(3-4):505–16. doi: 10.18632/genesandcancer.135 (PMC5489648; doi:10.18632/genesandcancer.135)
Supplement: Supplementary file 1 [file ganc-08-505-s001.pdf]

**Cdc25A is involved in ciclopirox-induced inhibition of cancer cell proliferation**

Tao Shen[1,2], Chaowei Shang[1,2], Hongyu Zhou[1], Yan Luo[1,3], Mansoureh Barzegar[1], Yoshinobu Odaka[1,2], Yang Wu[1,3], Shile Huang[1,2,*]

**Supplementary figures**

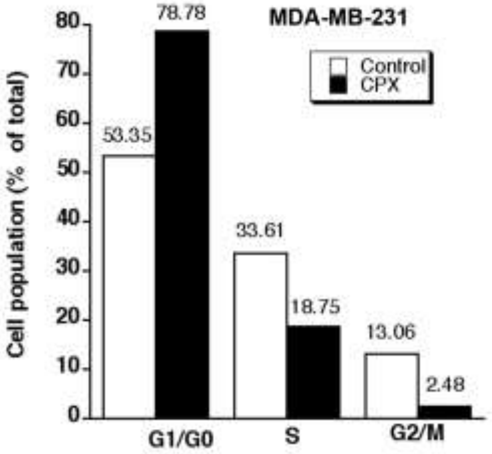


**Figure S1: CPX induces accumulation of MDA-MB-231 cells at $G_1$ phase of the cell cycle.** MDA-MB-231 cells were exposed to CPX (0 and 5 μM) for 24 h, followed by cell cycle analysis.

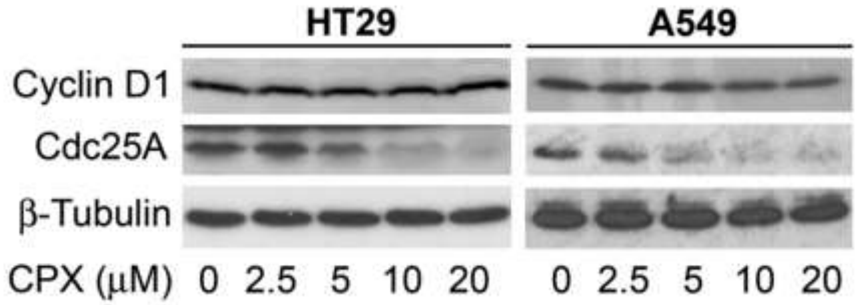


**Figure S2: CPX downregulates the cellular protein level of Cdc25A in tumor cells in a concentration-dependent manner.** HT29 and A549 cells were treated with CPX (0-20 μM) for 24 h, followed by Western blotting with indicated antibodies.

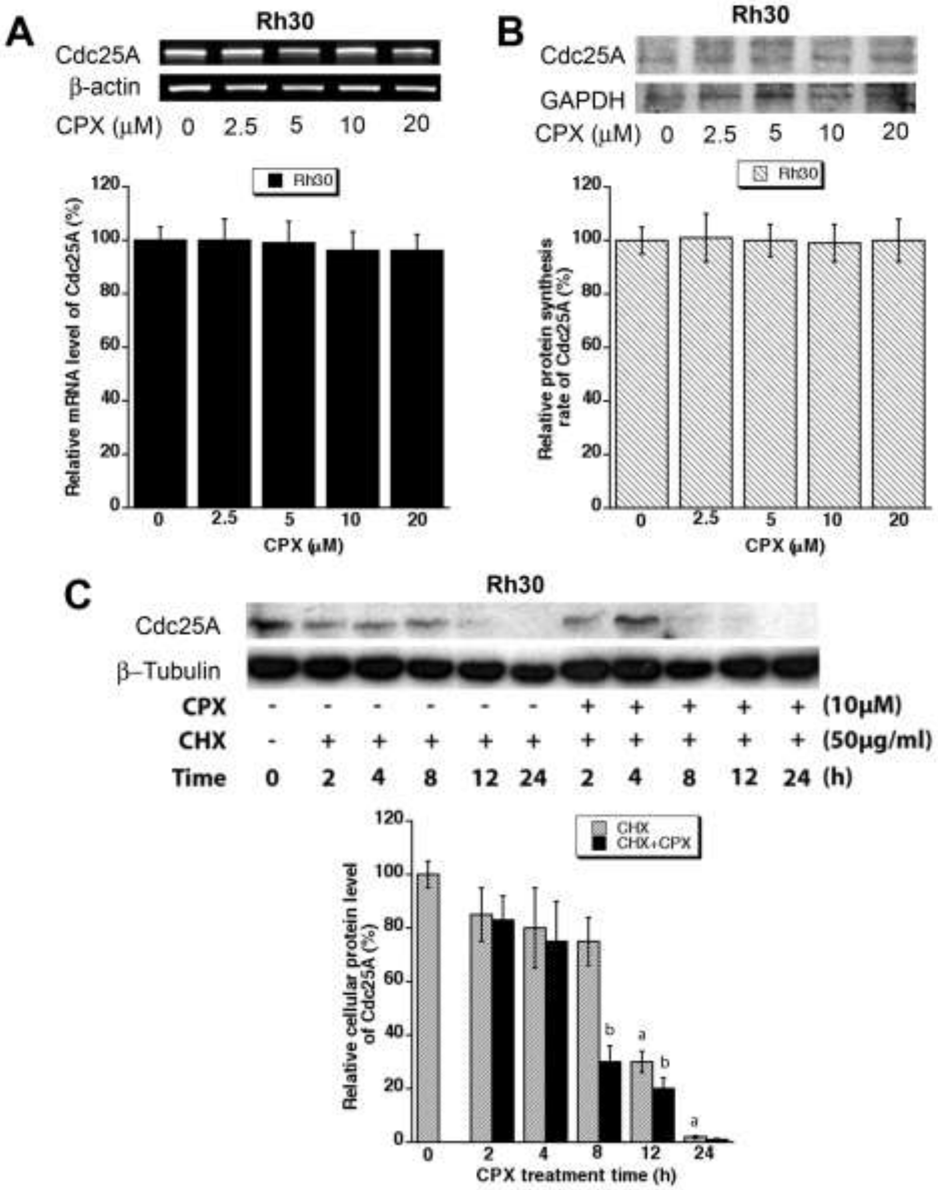


**Figure S3: CPX does not reduce Cdc25A mRNA level or protein synthesis significantly, but promotes Cdc25A protein degradation remarkably**. (A) Rh30 cells were treated with CPX (0-20 μM) for 24 h. Total mRNA was extracted, followed by RT-PCR. β-actin was used as internal control. (B) Rh30 cells were treated with CPX at 0-20 μM for 30 h, and then pulsed with $^{35}$S-Met/Cys for 6 h in the presence of CPX (0-20 μM). The proteins in the cell lysates were subjected to SDS-PAGE and transferred to a PVDF membrane, followed by autoradiography. GAPDH served as a control. (C) Rh30 cells were treated with 50 μg/ml cycloheximide (CHX), in the presence or absence of 10 μM CPX, for 0-24 h, followed by Western blotting with indicated antibodies. β-tubulin was used as a loading control. NIH Image J was used for semi-quantitative analysis of the intensities of the bands. Results are means ± SE and are pooled from three independent experiments. $^{a}P < 0.05$, difference *versus* control group. $^{b}P < 0.05$, difference *versus* CHX group.

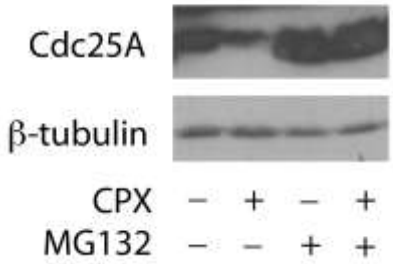


**Figure S4: Inhibition of proteasome with MG132 prevents CPX from reducing Cdc25A protein level.** MDA-MB-231 cells were treated with or without CPX (10 μM) for 24 h, in the presence or absence of MG132 (10 μM), followed by Western blotting with indicated antibodies.

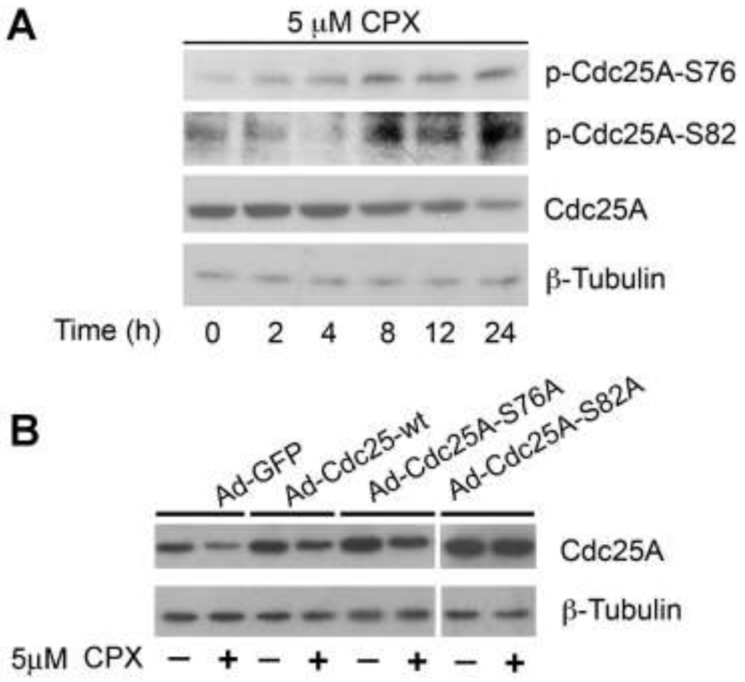


**Figure S5: CPX-induced Cdc25A phosphorylation contributes to its degradation.** (A) MDA-MB-231 cells were treated with CPX (5 µM) for 0-24 h, followed by Western blotting with indicated antibodies. (B) MDA-MB-231 cells, infected with indicated recombinant adenoviruses, were treated with or without CPX (5 µM) for 24 h, followed by Western blotting with indicated antibodies. β-tubulin was used as a loading control.

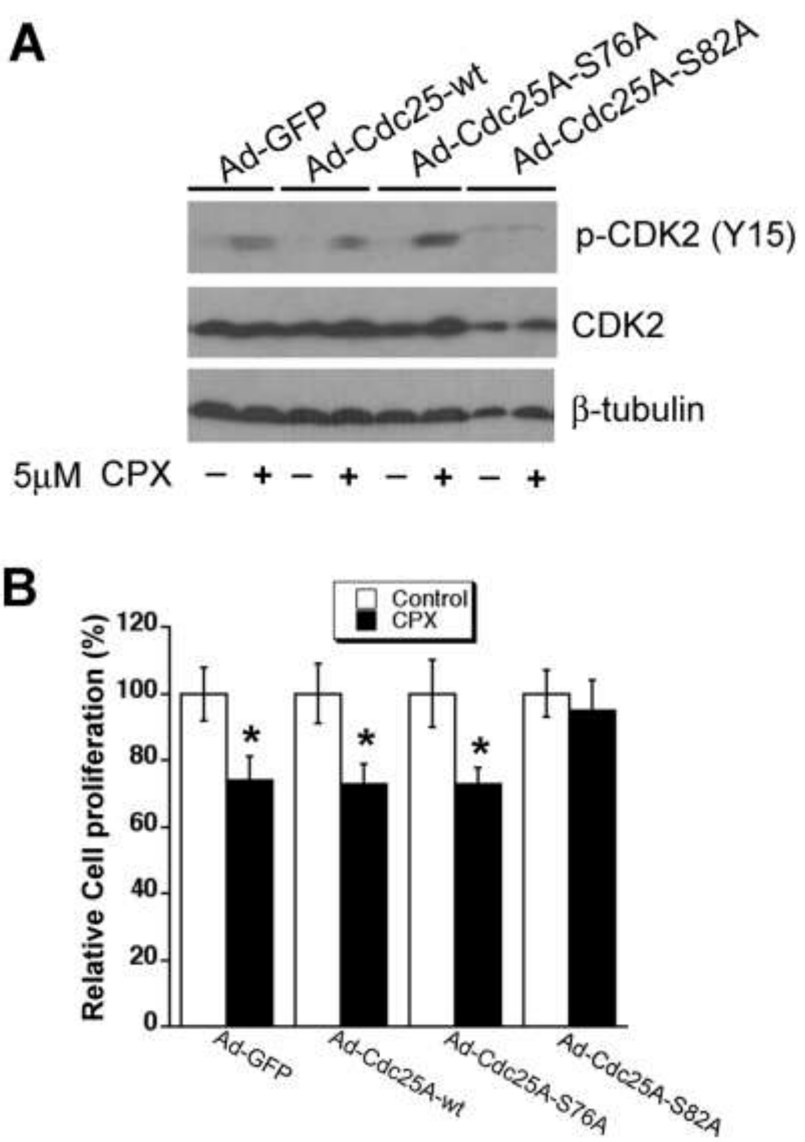


**Figure S6: Ectopic expression of Cdc25A mutant (S82A) renders resistance to CPX inhibition of cell proliferation.** (A, B) MDA-MB-231 cells, infected with indicated recombinant adenoviruses, were treated with 5 μM of CPX for 24 h, followed by Western blotting with indicated antibodies (A), and MTS assay (B). All data represent the means ± SE (n=3). *$P$< 0.05.
